# Developmental and light regulation of tumor suppressor protein PP2A in the retina

**DOI:** 10.18632/oncotarget.23351

**Published:** 2017-12-17

**Authors:** Ammaji Rajala, Yuhong Wang, Steven F. Abcouwer, Thomas W. Gardner, Raju V.S. Rajala

**Affiliations:** ^1^ Department of Ophthalmology, University of Oklahoma Health Sciences Center, Oklahoma City, Oklahoma, USA; ^2^ Department of Physiology, University of Oklahoma Health Sciences Center, Oklahoma City, Oklahoma, USA; ^3^ Department of Cell Biology, University of Oklahoma Health Sciences Center, Oklahoma City, Oklahoma, USA; ^4^ Dean McGee Eye Institute, University of Oklahoma Health Sciences Center, Oklahoma City, Oklahoma, USA; ^5^ Department of Ophthalmology and Visual Sciences, University of Michigan Medical School, Ann Arbor, Michigan, USA; ^6^ W.K. Kellogg Eye Center, University of Michigan Medical School, Ann Arbor, Michigan, USA

**Keywords:** anti-oncogene, protein phosphatase-2A, mechanistic target of rapamycin, retina, protein kinase C, Gerotarget

## Abstract

Protein phosphatases are a group of universal enzymes that are responsible for the dephosphorylation of various proteins and enzymes in cells. Cellular signal transduction events are largely governed by the phosphorylation of key proteins. The length of cellular response depends on the activation of protein phosphatase that dephosphorylates the phosphate groups to halt a biological response, and fine-tune the defined cellular outcome. Dysregulation of these phosphatase(s) results in various disease phenotypes. The retina is a post-mitotic tissue, and oncogenic tyrosine and serine/ threonine kinase activities are important for retinal neuron survival. Aberrant activation of protein phosphatase(s) may have a negative effect on retinal neurons. In the current study, we characterized tumor suppressor protein phosphatase 2 (PP2A), a major serine/ threonine kinase with a broad substrate specificity. Our data suggest that PP2A is developmentally regulated in the retina, localized predominantly in the inner retina, and expressed in photoreceptor inner segments. Our findings indicate that PKCα and mTOR may serve as PP2A substrates. We found that light regulates PP2A activity. Our studies also suggest that rhodopsin regulates PP2A and its substrate(s) dephosphorylation. PP2A substrate phosphorylation is increased in mice lacking the A-subunit of PP2A. However, there is no accompanying effect on retina structure and function. Together, our findings suggest that controlling the activity of PP2A in the retina may be neuroprotective.

## INTRODUCTION

Protein phosphatase 2 (PP2), commonly known as PP2A, is encoded by the PPP2CA gene in humans [1]. This enzyme is ubiquitously expressed, has conserved serine/threonine phosphatase activity with broad substrate specificity, and is able to dephosphorylate phosphorylation on serine and threonine residues on substrate proteins [2]. PP2A is a heterotrimeric protein phosphatase composed of structural A-, catalytic C-, and one of several regulatory B-subunits [3]. The formation of a heterotrimeric complex is dependent on the A-subunit, which serves as a scaffold for the other subunits to associate to form a holoenzyme [4]. Binding of the A-subunit to the catalytic C-subunit alters the phosphatase activity, even in the absence of a regulatory B-subunit [5]. Four classes of variable regulatory B-subunits have been identified in multicellular eukaryotes, with at least 16 members of these subfamilies: B (PR55), B’ (B56 or PR61), B” (PR72), and B”’ (PR93/PR110) [6]. Remarkable sequence conservation has been observed with C- and A-subunit sequences throughout eukaryotes, whereas more heterogeneous sequence conservation has been observed with B-subunits [6]. These B-subunits regulate the localization and specific activities of different heterotrimeric phosphatase holoenzymes *in vivo* [7]. The PP2A activity and subunit associations could also be regulated by other proteins and posttranslational modifications [2]. PP2A has been identified as a tumor suppressor for blood cancers, and mutations in the Aα-subunit (E64D) increased the incidence of cancers due to the lack of tumor-suppressing phosphatase activity [4]. Mutations in the A-subunit are defective in binding to the B-subunit or both the B- and C-subunits [8–10].

Our earlier studies suggest significantly reduced Rictor-bound mTOR phosphorylation (S2481) in diabetic rat retina [11]. mTOR activity has been shown to be regulated by PP2A in other cell types [12, 13]. However, there have been no studies of PP2A in the retina. Overexpression of mTOR has been reported in a variety of tumors [14]. Mutations inactivating PP2A A-subunits occur in human tumors [8–10]. A PP2A inhibitor, calyculin A, activates m-TORC2 in a concentration-dependent manner [13]. Further, PP2A contributes to endothelial cell death in response to high glucose [15]. PP2A is also hyperactive in an animal model of insulin resistance [16]. An elevated level of PP2A activity has been reported in retinal degeneration-1 mutant mice [17]. More recently, Regulated in Development and DNA Damage 1 (REDD1) protein has been shown to mediate PP2A-mediated dephosphorylation of Akt to repress mTORC1 [18]. These findings suggest that activation of PP2A in the retina may have adverse effects on the retina, and inhibition of this activity may promote neuron survival. In the present study, we examined the expression, activity, and inhibition of PP2A activity on retinal structure and function.

## RESULTS

### Immunohistochemical localization of PP2A in the developing retina

**Figure 1:**
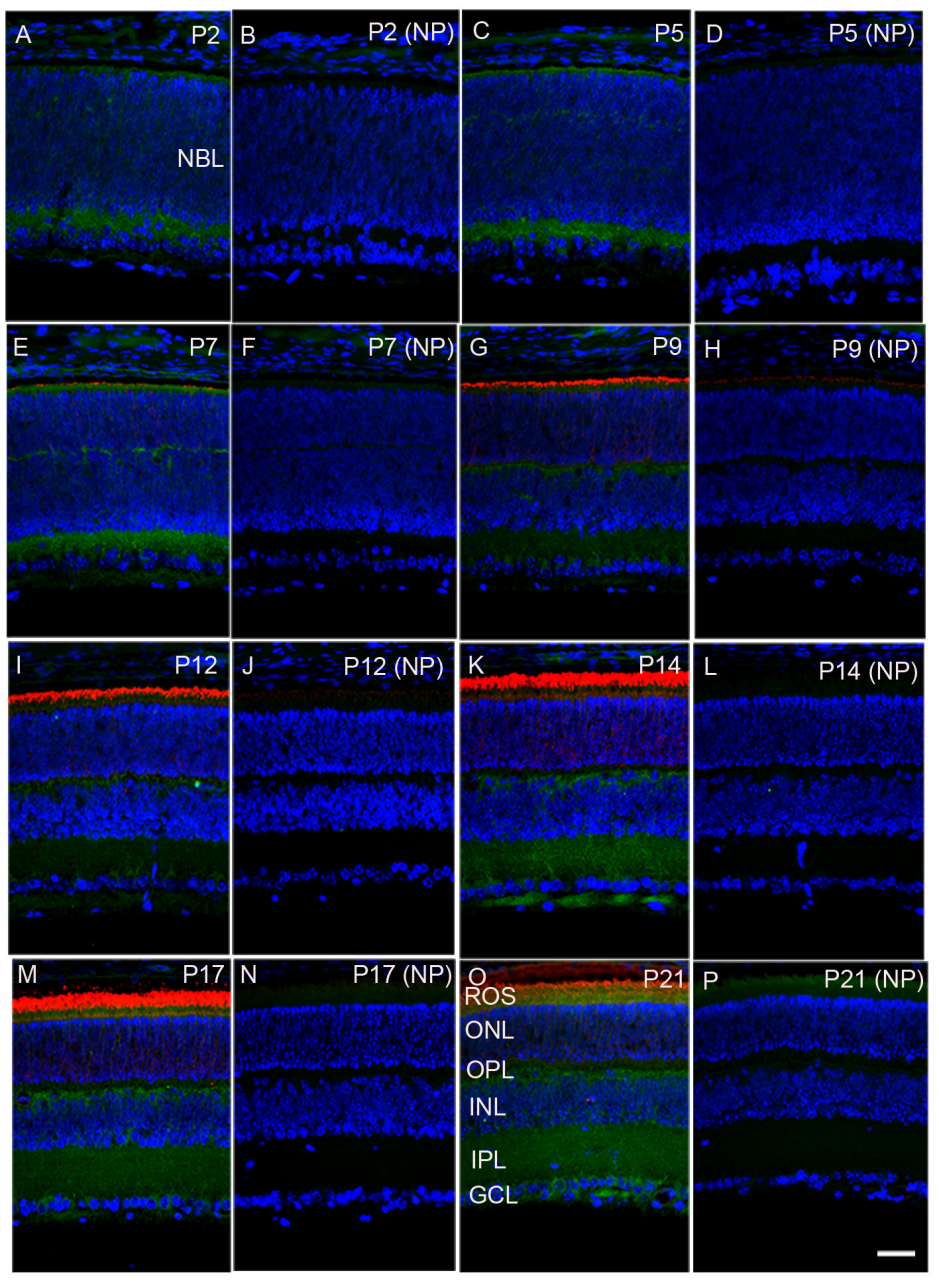
Developmental expression of PP2A in the retina Mouse retinal sections were prepared on postnatal day (P) 2, P5, P7, P9, P12, P14, P17, and P21 and were stained with PP2A (green) and rhodopsin (red). Panels **A, C, E, G, I, K, M**, and **O** are stained with primary antibodies, whereas panels **B, D, F, H, J, L, N**, and **P** are no-primary controls. NBL, neuoblastic layer; ROS, rod outer segments; ONL, outer nuclear layer; OPL, outer plexiform layer; INL, inner nuclear layer; IPL, inner plexiform layer; GCL, ganglion cell layer. Scale bar = 50 μm.

To investigate the expression of PP2A at different times during postnatal development, we carried out immunohistochemistry using PP2A antibody on mouse retinal sections on postnatal day 2 (P2), P7, P9, P12, P14, P17, and P21. At P2, PP2A expression was observed at both outermost choroidal and innermost vitreal boarders, presumably the inner plexiform layer of the retina (Figure 1A and 1B). At P5, the expression of PP2A was almost similar to P2, but some weak expression was observed in the outer neuroblastic layer where the cells are yet to be differentiated, and which gives rise to the outer plexiform layer (Figure 1C and 1D). At P7, rhodopsin expression was seen in the photoreceptor layer. PP2A expression was clearly noted in the photoreceptor layer, outer plexiform layer, inner plexiform layer, and some expression was also observed in the ganglion cell layer (Figure 1E and 1F). At P9 and P12, the rhodopsin expression was higher in the photoreceptor layer, while PP2A was expressed in the outer and inner plexiform layers. However, the expression of PP2A in the inner plexiform layer was weaker than that on P7 (Figure 1G-1J). The expression of PP2A was much higher in the inner plexiform and outer plexiform layers on P14 and P17 (Figure 1K-1N). On P21, the PP2A expression was higher in the outer plexiform, inner plexiform, and ganglion cell layers (Figure 1O, 1P). These results suggest that PP2A expression is developmentally regulated in the retina.

### Expression of PP2A in the retina under dark- and light-adapted conditions

**Figure 2:**
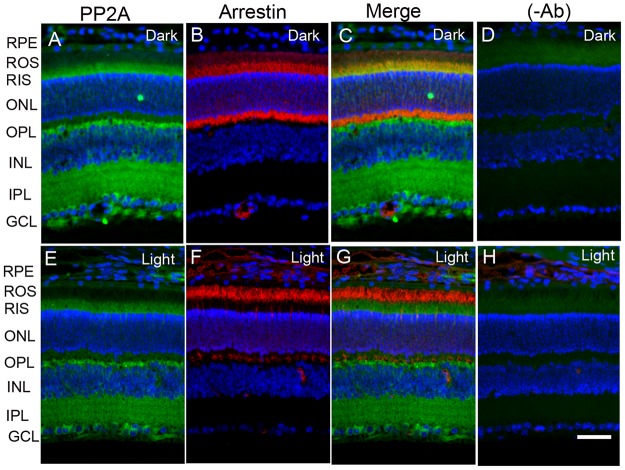
Immunofluorescence analysis of PP2A in mouse retina Prefer-fixed sections of dark- **A.**-**D.** and light-adapted **E.**-**H.** mouse retinas were stained for PP2A (A, E), arrestin (B, F), and DAPI (A-H). Panels C and G represent the merged images of PP2A and arrestin, whereas panels D and H represent the omission of primary antibodies. RPE, retinal pigment epithelium, ROS, rod outer segments; RIS, rod inner segments; ONL, outer nuclear layer; OPL, outer plexiform layer; INL, inner nuclear layer; IPL, inner plexiform layer; GCL, ganglion cell layer. Scale bar = 50 μm.

**Figure 3:**
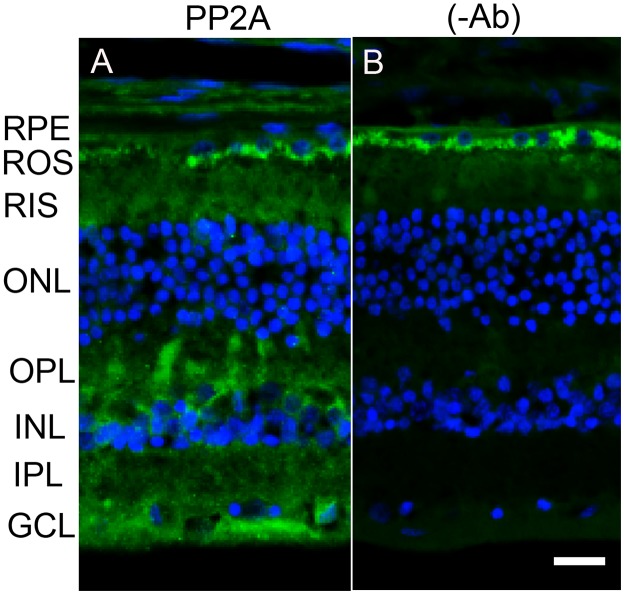
Immunofluorescence analysis of PP2A in postmortem human eye tissue Prefer-fixed sections of human retinas were stained for PP2A (green) and DAPI (blue) **A.** Panels **B.** represent the omission of primary antibody. RPE, retinal pigment epithelium, ROS, rod outer segments; RIS, rod inner segments; ONL, outer nuclear layer; OPL, outer plexiform layer; INL, inner nuclear layer; IPL, inner plexiform layer; GCL, ganglion cell layer. Scale bar = 50 μm.

**Figure 4:**
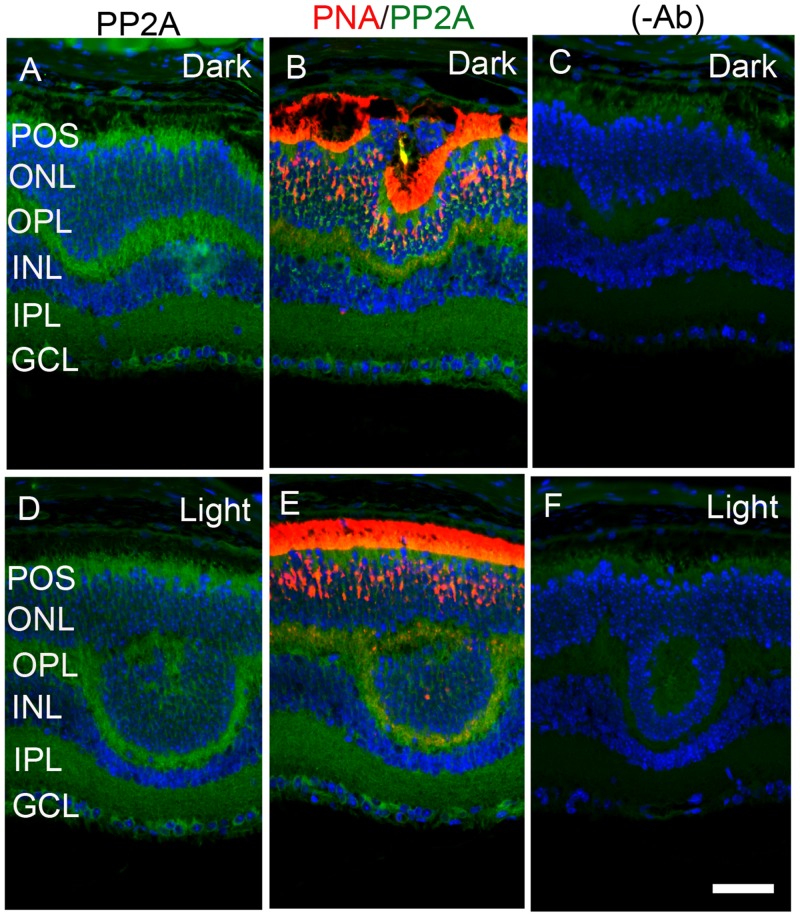
Immunofluorescence analysis of PP2A in cone-dominant *Nrl*^-/-^ mouse retina Prefer-fixed sections of dark- **A.**-**C.** and light-adapted **D.**-**F.** mouse retinas were stained for PP2A (A, B), PNA (B, E), and DAPI (A-F). Panels B and E represent the merged images of PP2A (green) and PNA (red), whereas panels C and F represent the omission of primary antibodies. POS, photoreceptor outer segments; RIS, rod inner segments; ONL, outer nuclear layer; OPL, outer plexiform layer; INL, inner nuclear layer; IPL, inner plexiform layer; GCL, ganglion cell layer. Scale bar = 50 μm.

The retina is a photosensitive tissue, due to the presence of light-absorbing photoreceptor cells and photosensitizing ganglion cells. The compartmentalization of several proteins in the retina is spatially regulated by dark- and light-adaptation. To determine whether light has an effect on the localization of PP2A, we stained mouse retinal sections prepared from dark- and light-adapted conditions and stained the sections with PP2A and rod arrestin antibodies. We used arrestin immunoreactivity to confirm the dark- and light-adaptation of the retina. In dark-adapted retina, the arrestin was localized to rod inner segments and the outer plexiform layer (Figure 2B). Upon light illumination, arrestin translocated to rod outer segments (Figure 2F). Our findings suggest that our mice were strictly dark- and light-adapted. Under these conditions, PP2A expression was predominantly localized to the rod photoreceptors, outer plexiform layer, inner plexiform layer and ganglion cell layer (Figure 2A and 2E), irrespective of either dark- or light-adapted conditions. In dark-adapted retina, PP2A co-localized with rod arrestin (Figure 2C). These results suggest that light does not play any role in the localization of PP2A. Figure 3 shows the localization of PP2A on post-mortem human eye tissue. The expression pattern of PP2A is comparable to mouse retina (Figure 3). To determine whether PP2A is expressed in cones, we took the advantage of *Nrl^-/-^* mice, serves as a mouse model for cone-dominant retina lacking rods due to the absence of *neural retina leucine zipper* (*Nrl)* transcriptional factor [19]. The *Nrl^-/-^* mouse retina has a characteristic feature of large undulations of the outer nuclear layer (ONL), commonly known as rosettes. These rosettes arise due to defects in the outer limiting membrane and delayed maturation of a subset of photoreceptors.^26^We stained dark- and light-adapted *Nrl^-/-^* mouse retinal section with PP2A and peanut agglutinin which labels cone segment sheets. Our results showed the expression of PP2A in cone photoreceptor and outer plexiform layers (Figure 4A-4F), and this expression remains same under both dark- and light- adapted conditions. These results suggest that PP2A is also expressed in cones.

### Biochemical characterization of PP2A in the retina

**Figure 5:**
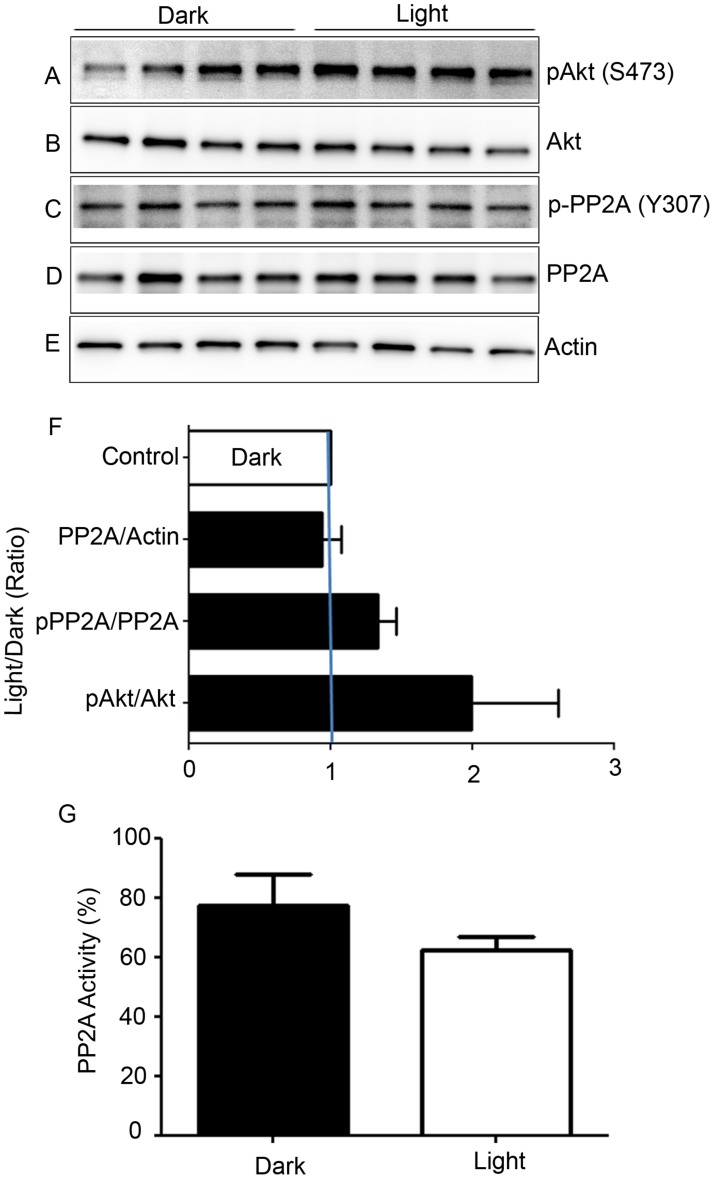
Biochemical characterization of PP2A, and phosphorylation state of PP2A-regulated protein Retinal lysates from dark- and light-adapted mice were subjected to immunoblot analysis with anti-pAkt (S473) **A.**, anti-Akt **B.**, p-PP2A (Y307) **C.**, anti-PP2A **D.**, and anti-actin **E.** antibodies. Densitometric analysis of pAkt, p-PP2A, and PP2A was performed in the linear range of detection, and absolute values were then normalized to Akt, PP2A, and actin **F.**. PP2A activity was measured from dark- and light-adapted mouse retinas **G**. as described in the Methods section. Data are mean + *SEM*, *n* = 4.

Retinal proteins from dark- and light-adapted wild-type mice were subjected to immunoblot analysis with anti-pAkt (S473), anti-Akt, anti-pPP2A (Y307), anti-PP2A, and anti-actin (Figure 5A-5E) antibodies. Akt is a known substrate of PP2A [20], and densitometric analysis of pAkt/Akt showed an increase in phosphorylation of Akt and PP2A under light-adapted conditions compared with dark-adapted conditions. However, this difference was not statistically significant (Figure 5F). Consistent with the phosphorylation of Akt, we found decreased PP2A activity under light-adapted conditions, although this difference was not statistically significant (Figure 5G). We found no difference in the levels of PP2A between dark- and light-adapted conditions (Figure 5D). The observation of decreased PP2A activity under light-adapted conditions could be due to light-dependent post-translational modification of PP2A. It was previously shown that tyrosine-307 phosphorylation on PP2A results in reduced activity [21, 22]. We found slightly increased phosphorylation of PP2A on Y307 under light-adapted conditions compared with dark-adapted conditions (Figure 5C). These findings suggest that PP2A may likely be post-translationally modified under light-adapted conditions.

### Light-dependent enhancement of PKCα and m-TOR phosphorylation in the retina

**Figure 6:**
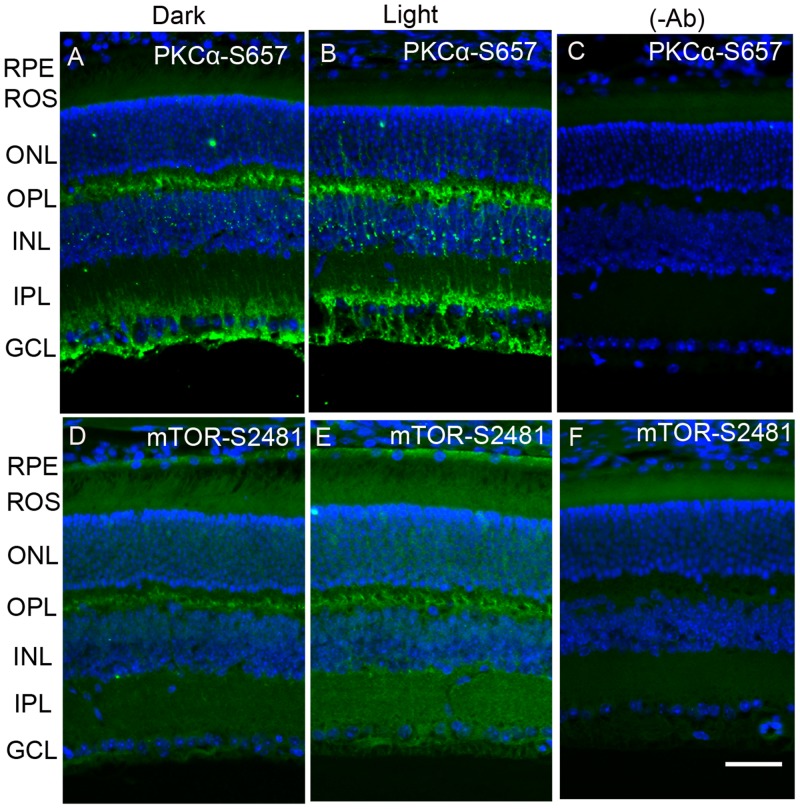
Immunofluorescence analysis of phosphorylation state of PKCα and m-TOR in dark- and light-adapted mouse retina Prefer-fixed sections of dark- **A.**, **D.** and light-adapted **B.**, **E.** mouse retinas were stained for PKCα-S657 (A, B) and mTOR-S2481 (D, E) antibodies. Panels **C.** and **F.** represent the omission of primary antibodies. Nuclear layers were stained with DAPI (blue). RPE, retinal pigment epithelium, ROS, rod outer segments; RIS, rod inner segments; ONL, outer nuclear layer; OPL, outer plexiform layer; INL, inner nuclear layer; IPL, inner plexiform layer; GCL, ganglion cell layer. Scale bar = 50 μm.

Existing literature suggests that PP2A regulates the phosphorylation state of PKCα [23] and m-TOR [12, 13]. Dark- and light-adapted mouse retinal sections were stained with anti-phospho-PKCα-S657 (p-PKCα) and anti-phospho-mTOR-S2481 (p-mTOR) antibodies. The results indicate that, in the dark, pPKC immunoreactivity was observed around the outer plexiform layer, below the inner plexiform layer and the ganglion cell layer. In the retina, PKCα is a known marker for rod bipolar cells. The observed PKCα immunoreactivity in the present study shows that rod bipolar cells are labelled with p-PKCα, which normally presents below the outer plexiform layer, and their dendrites end up in the inner plexiform and ganglion cell layers. Interestingly, PKCα phosphorylation is enhanced under light-adapted conditions (Figure 6A-6C). We also found mTOR phosphorylation in the outer plexiform layer. This phosphorylation was enhanced under light-adapted conditions and extended into the inner plexiform layer and ganglion cell layer (Figure 6D-6F). It appears that m-TOR phosphorylation may also take place in the rod bipolar cells. These observations suggest that PP2A substrates, PKCα, and mTOR phosphorylation could be regulated by light.

**Figure 7:**
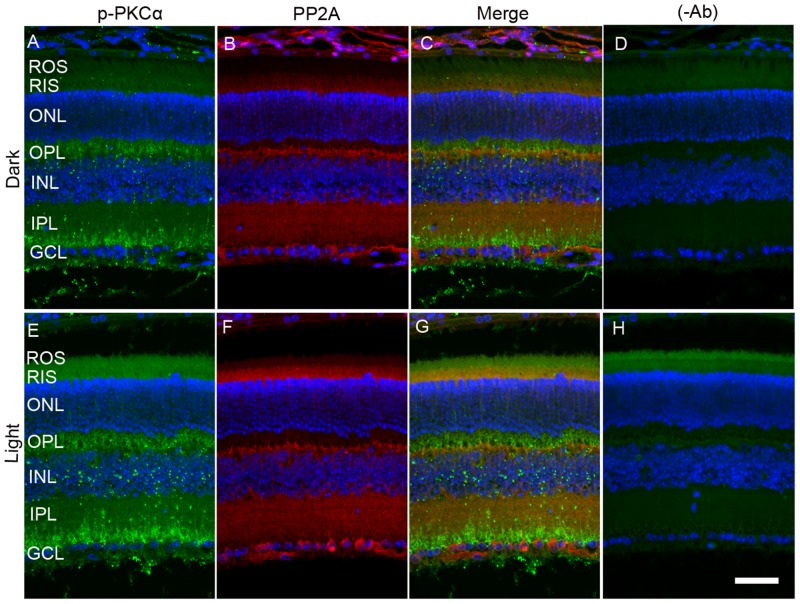
Immunofluorescence analysis of p-PKCα and PP2A in dark- and light-adapted mouse retina Prefer-fixed sections of dark- **A.**-**D.** and light-adapted **E.**-**H.** mouse retinas were stained for p-PKCα (green, A, E), PP2A (red, B, F), and DAPI (A-H). Panels C and G represent the merged images of p-PKCα and PP2A, whereas panels D and H represent the omission of primary antibodies. ROS, rod outer segments; RIS, rod inner segments; ONL, outer nuclear layer; OPL, outer plexiform layer; INL, inner nuclear layer; IPL, inner plexiform layer; GCL, ganglion cell layer. Scale bar = 50 μm.

When we co-stained dark- and light-adapted mouse retinal sections with p-PKCα and PP2A, we clearly observed increased p-PKCα immunoreactivity in light-adapted mouse retinas compared with dark-adapted mouse retinas (Figure 7A and 7E). PKCα is an authentic marker for rod bipolar cells; we observed a co-localization of PP2A and p-PKCα immunoreactivity in rod bipolar cells (Figure 7). The green (p-PKCα) signal observed in rod outer segment/rod inner segment regions under light-adapted condition is not a true signal (Figure 7E-7H), but resulted from autofluorescence, and could be observed even in the absence of p-PKCα primary antibody (Figure 7H).

**Figure 8:**
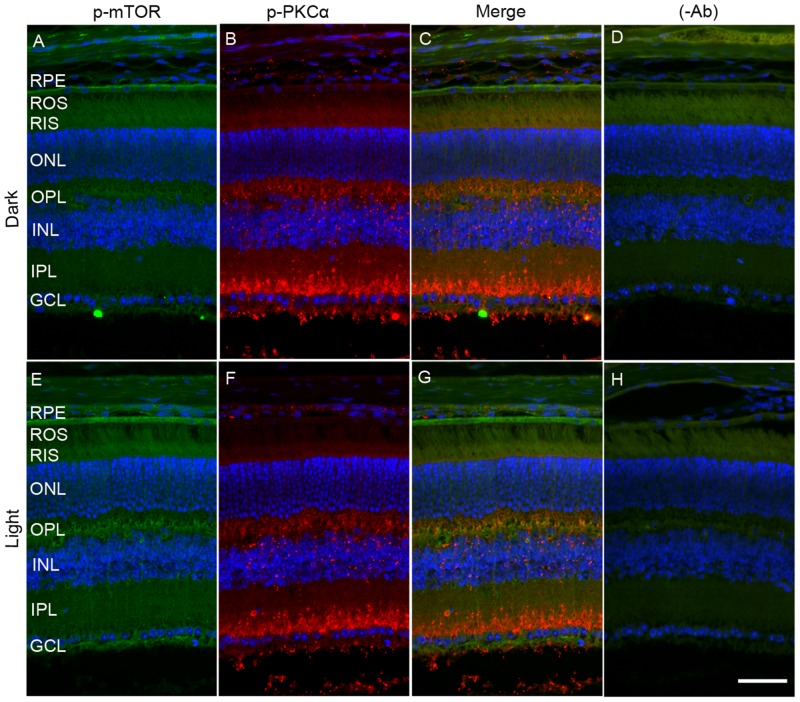
Immunofluorescence analysis of PKCα and mTOR in dark- and light-adapted mouse retina Prefer-fixed sections of dark- **A.**-**D.** and light-adapted **E.**-**H.** mouse retinas were stained for p-mTOR (green A, E), p-PKCα (red, B, F), and DAPI (A-H). Panels C and G represent the merged images of p-PKCα and p-mTOR, whereas panels D and H represent the omission of primary antibodies. ROS, rod outer segments; RIS, rod inner segments; ONL, outer nuclear layer; OPL, outer plexiform layer; INL, inner nuclear layer; IPL, inner plexiform layer; GCL, ganglion cell layer. Scale bar = 50 μm.

To determine whether mTOR is expressed in rod bipolar cells, we co-stained dark- and light-adapted mouse retinal sections with m-TOR and PKCα antibodies. We found that mTOR antibody did not work on prefer-fixed retinal sections (data not shown). Therefore, we stained the sections with p-mTOR (polyclonal antibody) and p-PKCα (goat polyclonal) antibodies. We could not use PKC antibody to co-label with p-mTOR antibody, as it was also a polyclonal antibody. Similar to data presented in Figure 6, panel E, we found increased phosphorylation of m-TOR in light-adapted retinas compared with dark-adapted retinas (Figure 8A and 8E). However, the p-mTOR signal that we observed in the RPE layer remained the same under both dark- and light-adapted conditions (Figure 8A, 8C, 8E and 8G). In this experiment, we found a co-localization of p-mTOR signal with p-PKCα in light-adapted retina, suggesting that m-TOR is expressed in rod bipolar cells (Figure 8G).

### Effect of photobleaching of rhodopsin on PP2A localization and its effect on substrate dephosphorylation

**Figure 9:**
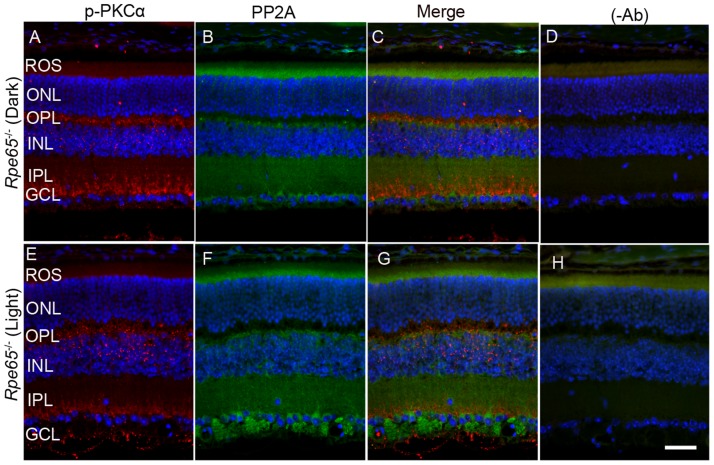
PKCα phosphorylation and PP2A expression in *Rpe65*^-/-^ mice Prefer-fixed sections of dark- **A.**-**D.** and light-adapted **E.**-**H.** *Rpe65^-/-^* mouse retinas were stained for p-PKCα (red, A, E), PP2A (green, B, F), and DAPI (A-H). Panels C and G represent the merged images of p-PKCα and PP2A, whereas panels D and H represent the omission of primary antibodies. ROS, rod outer segments; RIS, rod inner segments; ONL, outer nuclear layer; OPL, outer plexiform layer; INL, inner nuclear layer; IPL, inner plexiform layer; GCL, ganglion cell layer. Scale bar = 50 μm.

**Figure 10:**
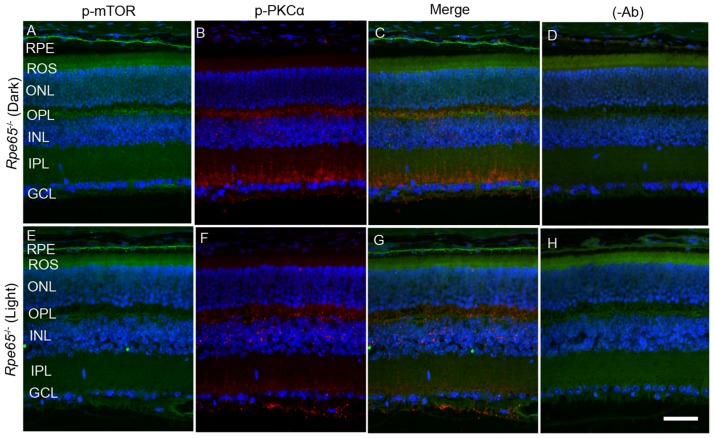
mTOR phosphorylation in *Rpe65*^-/-^ mice Prefer-fixed sections of dark- **A.**-**D.** and light-adapted **E.**-**H.** *Rpe65^-/-^* mouse retinas were stained for p-mTOR (green, A, E), p-PKCα (red, **B.**, **F.**, and DAPI (A-H). Panels C and G represent the merged images of p-mTOR and p-PKCα, whereas panels D and H represent the omission of primary antibodies. RPE, retinal pigment epithelium, ROS, rod outer segments; RIS, rod inner segments; ONL, outer nuclear layer; OPL, outer plexiform layer; INL, inner nuclear layer; IPL, inner plexiform layer; GCL, ganglion cell layer. Scale bar = 50 μm.

Retina is a photosensitive tissue, and the G-protein coupled receptor, rhodopsin activation, is an important physiological event that maintains the health of the retina. The chromophore 11-*cis*-retinal, generated from the retinal pigment epithelium cells adjacent to photoreceptor cells, is necessary for the light-absorption of rhodopsin and subsequent transmission of the nerve signal to other neurons for visual perception. Rhodopsin does not undergo photobleaching in mice lacking retinal pigment epithelium protein-65 (*Rpe65*) due to the absence of the regeneration of the chromophore [24]. To authenticate this mouse line, we examined the translocation of arrestin under dark- and light-adapted conditions. In *Rpe65^-/-^* mice, arrestin does not translocate to outer segments in a light-dependent manner and mislocalization occurs [25]. We found similar results in the trafficking of arrestin in this mouse line (Supplementary Figure 1). It was reported earlier that dark-adapted *Rpe65^-/-^* mice behaved as if they were light-adapted and activated transducin with unliganded opsin [26]. Consistent with these lines, we found increased PKCα phosphorylation in dark-adapted *Rpe65^-/-^* mouse retinas compared with light-adapted *Rpe65^-/-^* mouse retinas (Figure 9A and 9E). We also found an increased PP2A signal around the ganglion cell layer in light-adapted *Rpe65^-/-^* mouse retinas compared with dark-adapted *Rpe65^-/-^* mouse retinas (Figure 9B and 9F). We also noted increased mTOR phosphorylation in dark-adapted *Rpe65^-/-^* mouse retinas compared with light-adapted *Rpe65^-/-^* mouse retinas (Figure 10A and 10E). These findings suggest that photobleaching of rhodopsin regulates PP2A and subsequent dephosphorylation of its downstream targets. These experiments also indicate that the light response requires functional photoreceptor cells. This result excludes the possibility that the light-dependent activation occurs in photopigment-expressing ganglion cells, inner retinal neurons, or RPE [27, 28].

### Characterization of PP2A knock-in (PP2A-KI) mice and its effect on retinal structure, function and biochemistry

**Figure 11:**
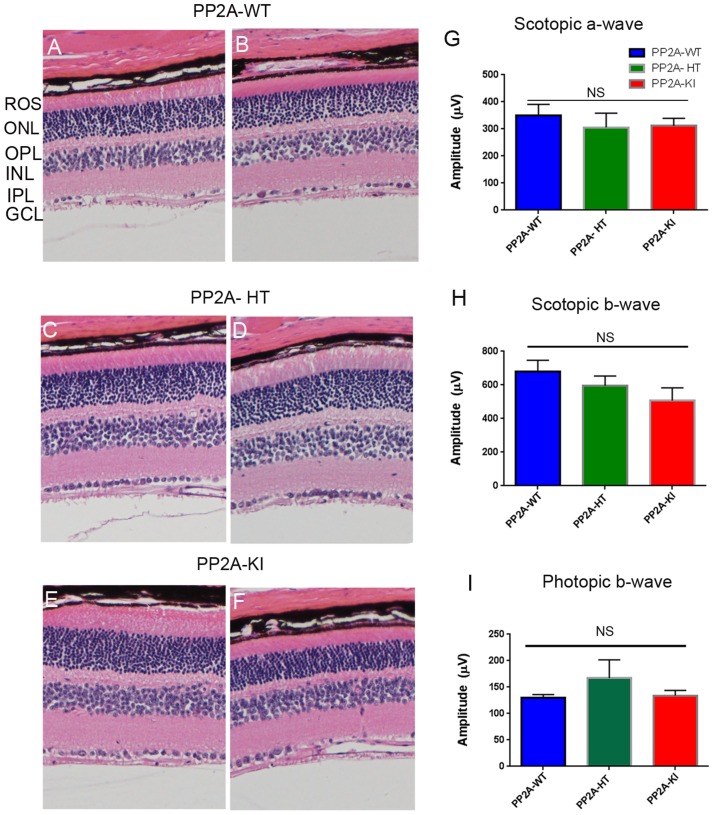
Structure and functional characterization of PP2A-KI mice Six-month-old PP2A- WT **A.**, **B.** PP2A-KI heterozygous **C.**, **D.**, and PP2A-KI homozygous **E.**, **F.** mouse retinal sections were stained with hematoxylin and eosin (H & E) and the morphology was examined. Examination of 6 retinas from each group did not reveal any structural differences in any of the retinal cells at the light microscope level. ROS, rod outer segments, ONL outer nuclear layer, OPL, outer plexiform layer, INL, inner nuclear layer, IPL, inner plexiform layer, GCL, ganglion cell layer. Two histological sections were presented for each genotype. Scotopic a-wave **G.**, scotopic b-wave **H.** and, photopic b-wave **I.** amplitudes of PP2A-WT, PP2A-KI heterozygous, and PP2A-KI homozygous mice were carried out at 6 months of age. Data are mean + *SEM*, *n* = 6.

**Figure 12:**
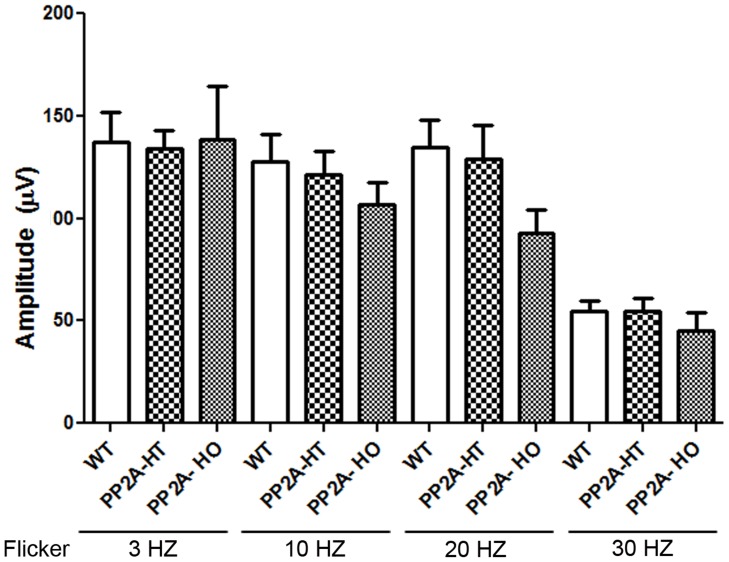
Quantitative analysis of flicker-flash ERG responses of PP2A-WT, PP2A-KI heterozygous, and PP2A-KI homozygous mice We measured cone function under conditions in which rod recovery is inhibited. The flicker responses at 3 HZ, 10 HZ, 20 HZ, and 30 Hz flicker were quantified for PP2A-WT, PP2A-KI heterozygous, and PP2A-KI homozygous mice. Data are mean + *SEM*, *n* = 6.

**Figure 13:**
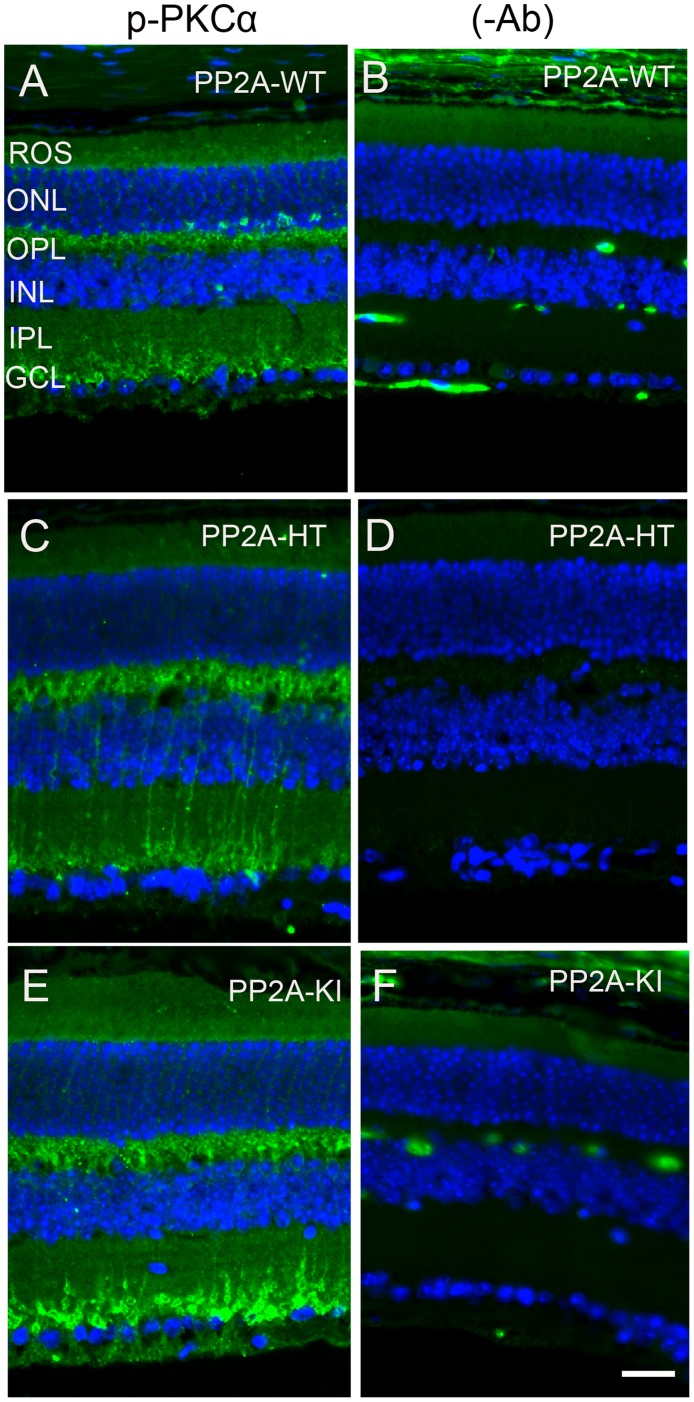
Increased PKCα phosphorylation in PP2A-KI mice Prefer-fixed sections of PP2A-WT **A.**, PP2A-KI heterozygous **C.**, and PP2A-KI homozygous **E.** mouse retinal sections were stained with p-PKCα antibody. Panels **B.**, **D.** and **F.** represent omission of primary antibody. Nuclear layers were stained with DAPI (A-F). ROS, rod outer segments; RIS, rod inner segments; ONL, outer nuclear layer; OPL, outer plexiform layer; INL, inner nuclear layer; IPL, inner plexiform layer; GCL, ganglion cell layer. Scale bar = 50 μm.

**Figure 14:**
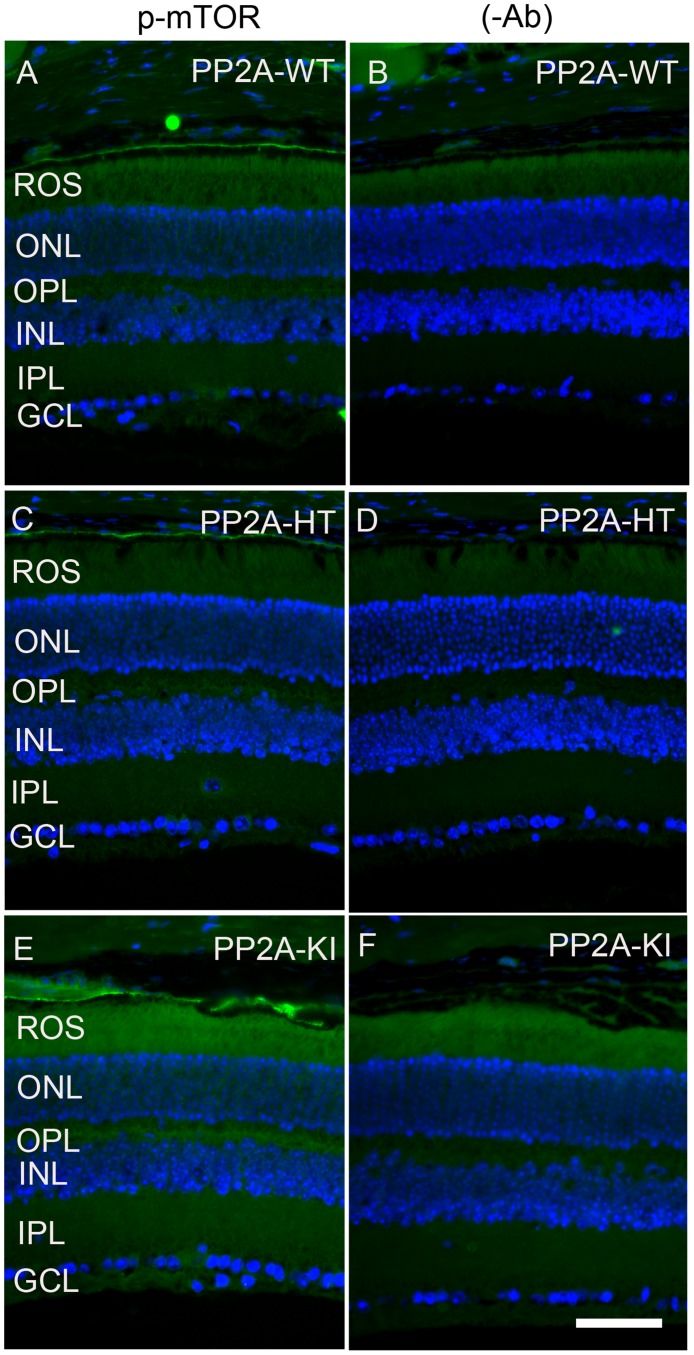
Increased mTOR phosphorylation in PP2A-KI mice Prefer-fixed sections of PP2A-WT **A.**, PP2A-KI heterozygous **C.**, and PP2A-KI homozygous **E.** mouse retinal sections were stained with mTOR antibody. Panels **B.**, **D.** and **F.** represent omission of primary antibody. Nuclear layers were stained with DAPI (A-F). ROS, rod outer segments; RIS, rod inner segments; ONL, outer nuclear layer; OPL, outer plexiform layer; INL, inner nuclear layer; IPL, inner plexiform layer; GCL, ganglion cell layer. Scale bar = 50 μm.

The PP2A holoenzyme consists of the scaffolding Aα-subunit, one of several B-subunits, and the catalytic Cα-subunit. It has been shown that mutations in Aα-subunit (E64D) in humans increased the incidence of cancers due to the lack of tumor-suppressing phosphatase activity [4]. These knock-in *PP2A-α-E64G* mice contain the amino acid mutation E64G in exon 3 of the A-α subunit gene (Ppp2r1α) of protein phosphatase 2A (*PP2A*). We re-derived this mouse line onto a C57Bl/6 background and bred out *rd1* and *rd8* mutations. Prefer-fixed 5-micrometer-thick sections of retinas from wild-type (C57Bl/6), PP2A-KI heterozygous, and PP2A-KI homozygous mice were cut along the vertical meridian and stained with hematoxylin and eosin to examine morphology. Rodents without any retinal degeneration show 11-12 rows of photoreceptor nuclei in the outer nuclear layer [29]. Morphology of the retina was not different among these genotypes, and the retinal cell integrity was well preserved, indicating that retinal cell viability was not affected in PP2A-KI mice (Figure 11A-11F). Electroretinography (ERG) recordings were used to assess and measure light driven rod- (scotopic a-wave, scotopic b-wave) and cone- (photopic b-wave) photoreceptor functional responses in 6-month-old wild-type, PP2A-KI-heterozygous, and PP2A KI-homozygous mice. No significant differences in ERG were found among these groups, indicating that mutation in the PP2Aα-subunit did not adversely affect the function of the retina (Figure 11G-11I). To evaluate the effect of phosphatase mutation in the PP2A on cone function under conditions when rod recovery was repressed, we carried out flicker-flash recordings. Our results indicate that PP2A-KI mutation did not suppress cone function under conditions in which rod recovery is inhibited (Figure 12). To test the hypothesis that PP2A regulates the phosphorylation of PKCα, retinal sections from wild-type, PP2A-KI heterozygous, and PP2A-KI homozygous mice were stained with anti-p-PKCα antibody. The results indicate the phosphorylation of PKCα in all genotypes (Figure 13A, 13C, and 13E); however, the phosphorylation of PKCα was enhanced in PP2A-KI homozygous mouse retinas (Figure 13E). These observations suggest that the state of PKCα phosphorylation is regulated by PP2A. We also found increased phosphorylation of m-TOR in PP2A-KI homozygous mice compared with wild-type mice (Figure 14). These experiments suggest that PP2A regulates the phosphorylation state of PKCα and m-TOR *in vivo*.

### Effect of PP2A on PDE6β-mediated retinal degeneration

**Figure 15:**
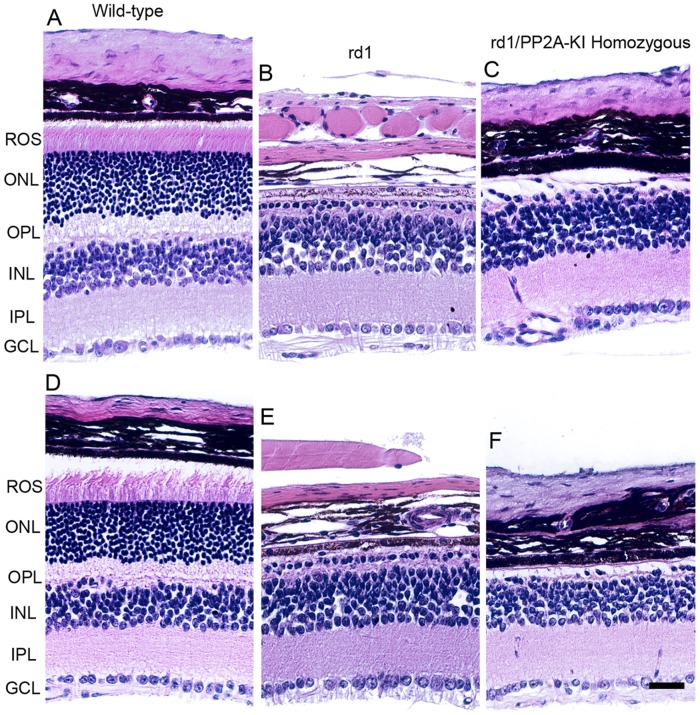
Effect of PP2A on PDE6β-mediated retinal degeneration One-month-old WT **A.**, **D.**, rd1 **B.**, **E.** and rd1/PP2A-KI **C.**, **E.** mouse retinal sections were stained with hematoxylin and eosin (H & E) and the morphology was examined. ROS, rod outer segments; RIS, rod inner segments; ONL, outer nuclear layer; OPL, outer plexiform layer; INL, inner nuclear layer; IPL, inner plexiform layer; GCL, ganglion cell layer. Scale bar = 50 μm.

Mutation in the photoreceptor cGMP phosphodiesterase 6β (*Pde6β*) gene (formally known as rd1), initially referred to as rodless mouse [30], undergo rapid photoreceptor degeneration beginning at postnatal day 9 [31, 32]. PP2A activity has been previously shown to be elevated in rd1 mouse retina [17]. We generated rd1/PP2A-KI homozygous mice and examined whether altering the PP2A activity prevents or slows down the rd1-mediated retinal degeneration. Our results show that rd1/PP2A-KI homozygous mice still show retinal degeneration (Figure 15). The retinal degeneration caused by rd1 mutation is rapid, and altering the PP2A activity is not able to prevent the retinal degeneration. Further studies are required to examine the effect of PP2A on slow retinal degenerative mouse models.

## DISCUSSION

Protein phosphorylation and dephosphorylation is one of the best understood mechanisms in cellular regulation. There are several protein kinases, but a limited number of protein phosphatases, that control cellular events [33]. Protein phosphatases are broadly classified into protein serine/threonine kinases and protein tyrosine kinases [33]. PP2A belongs to the protein threonine/serine kinase family and has board substrate specificity. In addition, PP2A is a tumor suppressor gene, has received a great deal of attention in the cancer field, and is mutated in several cancers [4, 9, 10].

Phosphatase and tensin homolog (*PTEN*) is a tumor suppressor protein that is also mutated in cancer [34]. Retinal neurons are post-mitotic cells, and, interestingly, ablation of PTEN in retinal degenerative mouse models promotes neuron survival [35]. Elevated levels of PP2A have been reported in mouse retinas undergoing retinal degeneration [17]. Protein tyrosine phosphatase-1B (PTP1B) has also been shown to be a tumor suppressor [36], and ablation or inhibition of its activity in the retina promotes retinal neuron survival under stress conditions [37]. These findings suggest a consensus that phosphatase activities must be tightly regulated in the retina in order to preserve phosphorylation on the target proteins.

There have been several studies on PP2A in the retina [17, 38], but none clearly demonstrated the developmental expression, localization and substrate specificities of the PP2A in the retina. In the present study, we characterized PP2A in terms of developmental expression, localization under dark- and light-adaption conditions, enzyme activities, substrate phosphorylation, and the functional consequences of the loss of phosphatase activity on retinal structure and function.

Our localization studies suggest that PP2A is predominantly expressed in inner retinal layers. We also observed expression in rod inner segments. This expression pattern remained the same under dark- and light-adapted conditions. However, the PP2A activity appeared to be inhibited under light-adapted conditions. We examined two protein substrates of PP2A, PKCα and m-TOR. The phosphorylation of both was enhanced under light-adapted conditions and also in mice deficient in PP2A activity. Our findings suggest that rhodopsin bleaching regulates the PP2A and subsequent dephosphorylation of its downstream targets.

Protein kinase C (PKC) family members play an important role in controlling numerous cellular processes, including gene expression, protein secretion, cell proliferation, and the inflammatory response [39]. The PKC family members are broadly classified into three groups based on the binding of second messengers [40]. The conventional PKC enzymes, including PKCα, PKCβ, and PKCγ, are commonly called cPKC isoforms, and their activation requires the binding of diacylglycerol (DAG), a phospholipid, and calcium [40, 41]. Novel PKC enzymes, including PKCδ, PKCε, PKCη, and PKCθ, are commonly known as nPKC isoforms and also require DAG [40, 41]. Atypical enzymes, such as PKCζ and PKCι/λ, are commonly called aPKC isoforms and do not require second messengers for aPKC activation [40, 41]. The enzyme phosphoinositide-dependent protein kinase-1 (PDK1) or a closely related kinase is accountable for PKC activation [42], and PKC activity is further regulated by phosphorylation [42].

In the current study, PKCα was exclusively expressed in rod bipolar cells, and its phosphorylation was light-and PP2A-dependent. For proper activation and termination of rod bipolar cell response, PKCα is essential [43]. It was reported earlier that diabetes causes defects in the rod signaling pathway, leading to decreased light-evoked rod bipolar cell inhibition and increased rod pathway output that provide a basis for the development of early diabetic visual defects [44]. Different PKC isoform (PKC-α, -β1/2, and PKC-δ)-mediated perturbations in vascular homeostasis have also been reported [41]. Clinical trials of PKCβ-isoform inhibitors have revealed some positive results for diabetic non-proliferative retinopathy, nephropathy, and endothelial dysfunction [41]. These observations suggest that PKC isoforms might play different roles in endothelial and neuronal cells, and that PP2A activity might also be differentially regulated in these cell types.

The mechanistic target of rapamycin (mTOR) activity has been shown to be regulated by PP2A [12, 13]. Our earlier studies showed reduced Rictor-bound S2481 m-TOR phosphorylation [11]. The current study revealed light-dependent activation of mTOR in the retina. Our findings also suggest the expression of mTOR in rod bipolar cells and increased phosphorylation of mTOR in PP2A activity-deficient mutant mice. Further studies are required to examine the role of PP2A in diabetes and m-TOR activation, in addition to understanding the role of m-TOR in bipolar cell functions. Tyrosine-307 phosphorylation on PP2A has been shown to inhibit PP2A activity [21, 22]. We observed increased phosphorylation of this residue in light-adapted retina. The Y307 phosphorylation on PP2A is regulated by insulin in other cell types [21, 22]. Experiments conducted with PP2A-KI mice clearly show an increased phosphorylation of PKC and m-TOR. It has been shown previously that PP2A contributes to endothelial cell death in response to high glucose [15], and is also hyperactive in an animal model of insulin resistance [16]. Since PP2A activity is involved in diabetes, the PP2A-KI mice will be a useful model with which to study the effect of diabetes on mTOR activity in PP2A-KI mice.

## MATERIALS AND METHODS

### Antibodies and reagents

Polyclonal p-mTOR-S2441, polyclonal pAkt (S473), polyclonal Akt, and polyclonal PP2A antibodies were obtained from Cell Signaling (Danvers, MA). Goat polyclonal p-PKCα-S657 antibody was procured from Santa Cruz Biotechnology (Dallas, TX). Polyclonal PKCα antibody was purchased from Sigma (St Louis, MO). Polyclonal p-PP2A-Y307 antibody was obtained from Thermo Fisher Scientific (Waltham, MA). DAPI stain used for nuclear staining and secondary antibodies were purchased from Invitrogen-Molecular Probes (Carlsbad, CA). Monoclonal anti-arrestin antibody was a kind gift from Dr. Paul Hargrave (University of Florida, Gainesville). All other reagents were of analytical grade and were procured from Sigma (St. Louis, MO). The PP2A immunoprecipitation phosphatase assay kit was purchased from EMD Millipore (Billerica, MA).

### Animals

All animals were treated in accordance with the *ARVO Statement for the Use of Animals in Ophthalmic and Vision Research* and the *NIH Guide for the Care and Use of Laboratory Animals.* The protocols were approved by the IACUC at the University of Oklahoma Health Sciences Center. Animals were born and raised in our vivarium and kept under dim cyclic light (40-60 lux, 12 h light/dark cycle). The *Rpe65^-/-^* mice were a kind gift from Dr. Jing-Xing Ma (University of Oklahoma Health Sciences Center, Oklahoma City). The *Nrl*^-/-^ mice were kindly provided by Dr. Anand Swaroop (NIH, Bethesda, MD). For light/dark experiments, mice were dark-adapted overnight. The next morning, half of the mice were exposed to normal room light (300 lux equivalent to 3000 R*/rods/sec) for 30 min [45]. Then, the eyes or retinas were harvested after CO_2_ asphyxiation. These tissues were subjected to biochemistry or immunohistochemistry. PP2A-E64D human mutation knock-in-mice (PP2A-KI mice) were re-derived onto a C57BL6 background from Jackson Labs. PP2A-E64D mice was originally derived in FVB/N background (Jackson Labs), and are homozygous for the Pdebrd1 mutation (formally known as rd1) in the cGMP-phosphodiesterase β-subunit.

### Preparation of tissue for paraffin sectioning using Prefer as a fixative

Mice were euthanized by CO_2_ asphyxiation and the eyeballs were placed in Prefer solution (Anatech Ltd, Battle Creek, MI) for 15 min at room temperature, followed by 70% ethanol overnight. The tissue was paraffin-embedded, and 5-μm-thick sections were cut and mounted onto slides. Sections were deparaffinized in 2-3 changes of xylene (10 minutes each) and hydrated in 2 changes of 100% ethanol for 3 minutes each, 95% and 80% ethanol for 1 minute each, and then rinsed in distilled water. The slides were washed three times in 1X PBS containing 0.1% Triton-X 100, blocked with horse serum for 1 h, and primary antibody was added overnight at 4°C. For fluorescent detection, slides were incubated with a mixture of Texas-red-anti-mouse and FITC-anti-rabbit antibodies (Vector Laboratories, Burlingame, CA), each diluted 1:200 in PBS with 10% horse serum. After incubation for 1 h at room temperature, the slides were washed with PBS and cover-slipped in 50% glycerol in PBS. Antibody-labeled complexes were examined on a Nikon Eclipse E800 microscope equipped with a digital camera, and images were captured using Metamorph (Universal Imaging, West Chester, PA) image analysis software. All images were captured using identical microscope and camera settings so that intensities of the digital images reflected antibody binding.

### Immunoblot analysis

Retinas were homogenized in a lysis buffer containing 1% Triton X-100, 137 mM NaCl, 20 mM Tris-HCl (pH 8.0), 10% glycerol, 1 mM EGTA, 1 mM MgCl_2_, 1 mM phenylmethylsulfonyl fluoride, 0.2 mM Na_3_VO_4_, 10 μg/ml leupeptin, and 1 μg/ml aprotinin [46]. Insoluble material was removed by centrifugation at 17,000 x g for 20 min at 4°C, and the protein concentrations of the solubilized proteins were determined with the Bicinchoninic Acid reagent according to the manufacturer's instructions (Pierce Biotechnology, Rockford, IL). Proteins were resolved by 10% or gradient (4-20%) SDS-PAGE and transferred to nitrocellulose membranes. The blots were washed twice for 10 min with TTBS (20 mM Tris-HCl at pH 7.4, 100 mM NaCl and 0.1% Tween-20) and blocked with either 5% bovine serum albumin or non-fat dry milk powder (Bio-Rad) in TTBS for 1 h at room temperature. Blots were then incubated with anti-pAkt (S473) (1:1000), anti-Akt (1:1000), anti-PP2A (1:1000), anti-p-PP2A (Y307) (1:1000), and anti-actin (1:1000) antibodies overnight at 4°C. After the primary antibody incubations, immunoblots were incubated with HRP-linked secondary antibodies (mouse or rabbit or goat) and developed by enhanced chemiluminescence, according to the manufacturer's instructions.

### PP2A phosphatase activity

Two freshly harvested retinas were homogenized in 200 μl of homogenizing buffer [20 mM imidazole-HCl, 2 mM EDTA, 2 mM EGTA, pH 7.0 with 10 μg/ml each of aprotinin, leupeptin, pepstatin, 1 mM benzamidine, and 1 mM phenylmethylsufonyl fluoride (PMSF)]. The homogenate was centrifuged at 2000 x g for 5 min at 4°C. One hundred micrograms of protein was incubated with 4 μg of anti-PP2A, C-subunit, clone ID6, followed by 25 μl of protein-A agarose beads. We brought the volume to 500 μl with phosphatase assay buffer [50 mM Tris-HCl, pH 7.0, 100 μM CaCl_2_] and incubated the tubes for 2 hrs at 4°C with constant rocking. Then, we washed the beads three times with 700 μl of TBS buffer [50 mM Tris-HCl, pH 7.6, 150 mM NaCl], followed by one wash with 500 μl of phosphatase assay buffer. To assay tubes, we added 60 μl (750 μM) of phosphopeptide (K-R-pT-I-R-R) and 20 μl of phosphatase assay buffer. Then, the tubes were incubated for 10 min at 30°C in a shaking incubator. The reaction products were centrifuged briefly. Then, we transferred 25 μl into each well of the half-volume flat bottom microtiter plate, added 100 μl of Malachite Green Phosphate detection solution, and read the absorbance at 650 nm after 10 min.

### Electroretinography

Flash ERGs were recorded with the Diagnosys Espion E2 ERG system (Diagnosys, LLC, Lowell, MA). Mice were maintained in total darkness overnight and prepared for ERG recording under dim red light. They were anesthetized with ketamine (100 mg/kg body weight) and xylazine (6 mg/kg body weight) intramuscularly. One drop of 10% (v/v) phenylephrine was applied to the cornea to dilate the pupil, and one drop of 0.5% (v/v) proparacaine HCl was applied for local anesthesia. Mice were kept on a heating pad at 37°C during recordings. A gold electrode was placed on the cornea, a reference electrode was positioned in the mouth, a ground electrode was placed on the foot, and mice were placed inside a Ganzfeld illuminating sphere. Responses were differentially amplified, averaged, and stored. For the assessment of rod photoreceptor function (scotopic ERG), five strobe flash stimuli were presented at flash intensities at −2.3, −1.3, 0.7, and 2.7 log cd·s/m^2^. The amplitude of the a-wave was measured from the prestimulus base line to the a-wave trough. The amplitude of the b-wave was measured from the trough of the a-wave to the peak of the b-wave. For the evaluation of cone function (photopic ERG), a strobe flash stimulus (3.3 log cd·s/m^2^) was presented to dilated, light-adapted (5 min at 1.7 log cd·s/m^2^) mice. The amplitude of the cone b-wave was measured from the trough of the a-wave to the peak of the b-wave. To evaluate cone function under conditions in which rod recovery is suppressed, flicker-flash ERG recordings were measured. Mice were placed under a steady adapting field of 1.7 log cd·s/m^2^ for at least 7 min. A single flash of 3.3 log cd·s/m^2^ was presented under the same adapting field to elicit a maximal cone response. Cone responses were further evaluated by presenting 1.2 log cd·s/m^2^ stimuli flickering at frequencies of 3, 10, 20, and 30 Hz under the same adapting field.

### Statistical analysis

One-way ANOVA and post-hoc statistical analysis using Bonferroni's pairwise comparisons were used to determine statistical significance (*p* < 0.05).

## SUPPLEMENTARY MATERIALS FIGURE


