# Supplementary material for: Developmental and light regulation of tumor suppressor protein PP2A in the retina
Source: Oncotarget. 2017 Dec 17;9(2):1505–23. doi: 10.18632/oncotarget.23351 (PMC5788578; doi:10.18632/oncotarget.23351)
Supplement: Supplementary file 1 [file oncotarget-09-1505-s001.pdf]

Developmental and light regulation of tumor suppressor protein PP2A in the retina

SUPPLEMENTARY MATERIAL

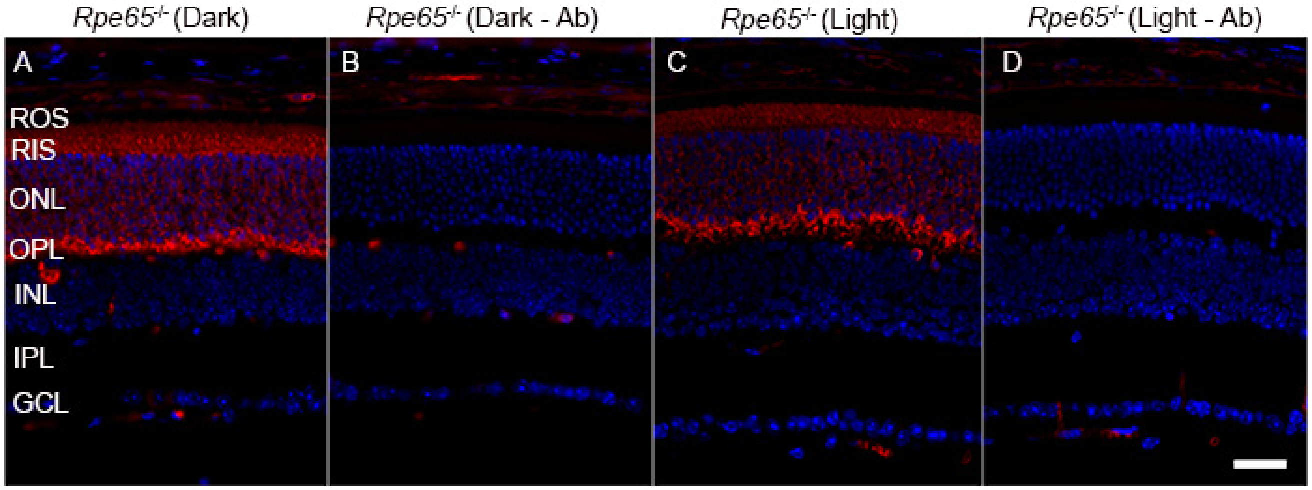

Supplementary Figure 1: Localization of rod arrestin in dark- and light-adapted Rpe65$^{-/-}$ mice. Prefer-fixed sections of dark- (**A, B**) and light-adapted (**C, D**) Rpe65$^{-/-}$ mouse retinas were stained for rod arrestin (**A, C**) and DAPI (**A-D**). Panels **B** and **D** represent the omission of primary antibody. ROS, rod outer segments; RIS, rod inner segments; ONL, outer nuclear layer; OPL, outer plexiform layer; INL, inner nuclear layer; IPL, inner plexiform layer; GCL, ganglion cell layer. Scale bar = 50 µm.
